# Prevalence, perceptions, and consequences of substance use in medical students

**DOI:** 10.1080/10872981.2017.1392824

**Published:** 2017-10-26

**Authors:** Erin E. Ayala, Destiny Roseman, Jeffrey S. Winseman, Hyacinth R.C. Mason

**Affiliations:** ^a^ Department of Counseling Psychology, St. Mary’s University of Minnesota, Minneapolis, MN, USA; ^b^ Department of Medical Education, Albany Medical College, Albany, NY, USA; ^c^ Department of Psychiatry, Albany Medical Center, Albany, NY, USA; ^d^ Department of Medical Education, Albany Medical College, Albany, NY, USA

**Keywords:** Medical students, medical education, substance use, alcohol, health

## Abstract

**Background:** Research regarding the health and wellness of medical students has led to ongoing concerns regarding patterns of alcohol and drug use that take place during medical education. Such research, however, is typically limited to single-institution studies or has been conducted over 25 years ago.

**Objective:** The objective of the investigation was to assess the prevalence and consequences of medical student alcohol and drug use and students’ perceptions of their medical school’s substance-use policies.

**Design:** A total of 855 medical students representing 49 medical colleges throughout the United States participated in an online survey between December 2015 and March 2016.

**Results:** Data showed that 91.3% and 26.2% of medical students consumed alcohol and used marijuana respectively in the past year, and 33.8% of medical students consumed five or more drinks in one sitting in the past two weeks. Differences in use emerged regarding demographic characteristics of students. Consequences of alcohol and drug use in this sample of medical students included but were not limited to interpersonal altercations, serious suicidal ideation, cognitive deficits, compromised academic performance, and driving under the influence of substances. Forty percent of medical students reported being unaware of their medical institution’s substance-use policies.

**Conclusions:** Findings suggest that substance use among medical students in the US is ongoing and associated with consequences in various domains. There is a lack of familiarity regarding school substance-use policies. Although there has been some progress in characterizing medical student alcohol use, less is known about the factors surrounding medical students’ use of other substances. Updated, comprehensive studies on the patterns of medical student substance use are needed if we are to make the necessary changes needed to effectively prevent substance-use disorders among medical students and support those who are in need of help.

## Introduction

Substance use is a major public health issue affecting the health and well-being of millions of Americans [1]. Medical students follow general young adult patterns and are not exempt from the consequences of substance use, which may include injuries, work and social impairment, violence, risky sexual behavior, cardiovascular disease, cancer, and death [1–3]. Studies assessing the prevalence of increased substance use among medical students in the United States (US) suggest that as many as 58% of medical students may binge drink monthly [4], one in three medical students have used illicit drugs in the past year [5], use typically begins during high school or college [6], and that alcohol and marijuana are the most commonly misused substances [5]. Although many studies examining medical student substance use have been published in recent years, the most comprehensive research in this area was conducted over 25 years ago [7].

New data reflecting the wide ranging patterns of alcohol and drug use in medical students and the sequelae of substance use that more accurately reflects current use by medical students in the US has yet to be compiled [8]. Researchers have suggested that substance use in medical school may be the root of the ongoing problem of increased substance use in practicing physicians [8–10]. Hence, identifying the rates and risks of substance use and the social patterns that promote or inhibit use during medical education is an increasingly important task. To address this gap, the purpose of our investigation was to (1) assess the prevalence of medical student alcohol and drug use; (2) determine the self-reported consequences of medical student substance use; and (3) assess perceptions of medical school substance-use policies.

## Methods

### Participants

A total of 855 students responded to the alcohol and drug-related questions in the survey. Students ranged in age from 20 to 45 and had a mean age of 25.64 (*SD *= 3.30). Students in the sample represented first- (30.6%, *n* = 262), second- (26.2%, *n* = 224), third- (21.6%, *n* = 185), and fourth- (20.0%, *n *= 171) years of training. There were more female (62.5%, *n *= 534) than male (35.6%, *n *= 304) respondents. The majority of students were Caucasian (73.9%, *n *= 656), followed by Asian (8.1%, *n* = 69), Hispanic (6.8%, *n* = 58), African American (4.3%, *n* = 37), Bi/Multiracial (3.6%, *n* = 31), and Native American (0.5%, *n* = 4). Fourteen percent of students identified as being a first generation college graduate (14.4%, *n* = 123). See Table 1 for additional information.

**Table 1.** Demographic characteristics of the sample.

	*n*	%	
*Biological sex*			
Female	534	62.5	
Male	304	35.6	
*Year*			
MS1	262	30.6	
MS2	224	26.2	
MS3	185	21.6	
MS4	171	20.0	
*Race/ethnicity*			
Caucasian	632	73.9	
Asian	69	8.1	
African American	37	4.3	
Hispanic	58	6.8	
Native American	4	0.5	
More than one race	31	3.6	
*Sexual orientation*			
Heterosexual/straight	755	88.3	
Gay or lesbian	21	2.5	
Bisexual	42	4.9	
Pansexual	10	1.2	
Queer/fluid	6	0.7	
Asexual	4	0.5	
*Marital status*			
Single	539	63.0	
Living with partner	80	9.4	
Married	148	17.3	
Engaged	67	7.8	
Separated/divorced	9	1.1	
*First generation college graduate*			
Yes	123	14.4	
No	718	84.0	
	*M*	*SD*	*Range*
*Age*	25.64	3.30	20–45

### Measures

#### Core alcohol and drug survey

We measured occurrence rates, perceptions, and consequences resulting from substance use using the Core Alcohol and Drug Survey [11]. This tool has been used extensively with undergraduate students [12], graduate students [13], and first-year law, dental, and medical students [14]. The survey was normed on a sample of 58,625 students at more than 800 campuses throughout the US [11].

The Core Alcohol and Drug Survey [11] includes 39 questions regarding frequency of alcohol and drug use in the past month and year, at risk drinking in the last two weeks, average drinks per week, perceptions regarding the institution’s alcohol and drug policies, and consequences of use in the past year. Participants respond to items on a Likert-type scale for questions regarding use of substances in the past-month (1 = *Never* to 7 = *Every day*) and past-year (1 = *Never*, 6 = *Ten or more times*). We excluded questions regarding frequency of use in residence halls and participation in athletics and college fraternities or sororities. Internal reliability for the scale was good (*α* = 0.80).

#### Demographic questionnaire

Participants were asked to record their age, gender, race, sexual orientation, year in medical school, name of medical school, and first generation student status. All items were optional and students were informed that demographic data would be used only in aggregate form.

### Procedure

From December 2015 to March 2016, we used a respondent driven sampling method [15] to invite medical students to participate in a confidential, IRB-approved online survey on medical student stress, health, and wellness. We distributed emails via professional listservs, social media, medical organizations, and to all Association of American Medical Colleges (AAMC) institutions within the US. The email provided information about the survey and invited administrators to distribute the email to medical students at their respective institutions. Of the 137 AAMC institutions contacted in the US, 14 schools in 11 states and the District of Columbia confirmed that they distributed the email to their medical students. This respondent driven sampling technique, recommended for special and geographically diverse populations [15], encourages those in the target population to distribute the survey to others in their social network. The survey subsequently reached US medical students from 49 medical schools in 29 states and the District of Columbia. The variables in the dataset that pertained to substance use patterns, consequences, and perceptions of school policies were used for this investigation.

### Statistical analysis

Responses were first examined for missing values, outliers, and assumptions of linearity, normality, and homoscedasticity [16]. Data were missing at random per Little’s Missing Completely at Random test, χ^2^ (36,393) = 999.912, *p* = 1.000 [17]. We performed descriptive analyses to obtain demographic information for participants, frequency of alcohol and drug use, consequences associated with alcohol and drug use, and perceptions associated with their respective medical institution’s alcohol and drug policies.

## Results

### Prevalence

**Figure 1.**
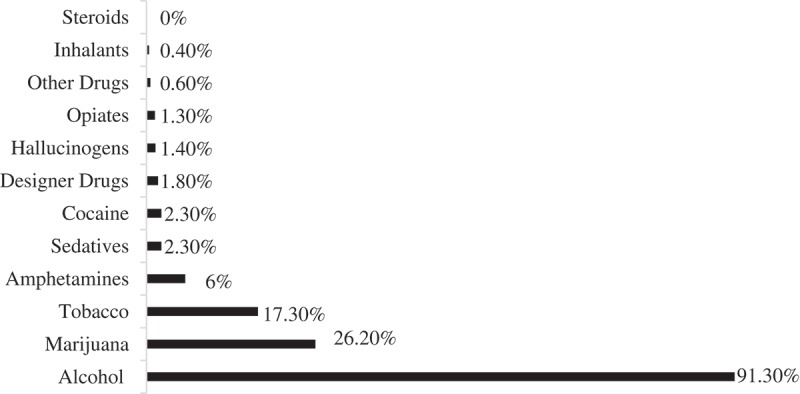
Self-reported substance use in the past year.

Prevalence rates for the 12 substances surveyed are displayed in Table 2 and Figure 1. Most students reported consuming alcohol in the past year (91.3%, *n *= 781), and most stated they preferred to have alcohol available at parties (81.4%, *n* = 696). In addition to alcohol, marijuana and tobacco were the most common substances used. Co-occurrence of marijuana and tobacco use was infrequent with 7.6% and 2.7% of medical students reporting the use of both substances in the past year and month, respectively. Sixteen percent of medical students (*n *= 136) stated they would prefer to have drugs other than alcohol available at parties.

**Table 2.** Descriptive statistics for alcohol and drug use in past 30 days.

	%	*M*^a^	*SD*
Alcohol	85.2	3.06	1.39
Drinks/week^b^	–	2.67	3.25
At risk drinking^c^	33.8	1.53	0.88
Marijuana	11.7	1.22	0.78
Tobacco	6.8	1.12	0.59
Amphetamines	4.4	1.17	0.89
Sedatives	1.3	1.03	0.37
Designer drugs	0.4	1.00	0.09
Cocaine	0.5	1.00	0.07
Other drugs	0.02	1.00	0.05
Steroids	0.01	1.00	0.04
Hallucinogens	0.1	1.00	0.04
Opiates	0.5	1.07	0.06
Inhalants	0.2	1.00	0.08

% = Percentage of medical students who consumed substance, M = Mean, SD = Standard deviation.

^a^1 = Never, 2 = 1–2 days, 3 = 3–5 days, 4 = 6–9 days.

^b^M = number of drinks per week.

^c^M = 5+ drinks in one sitting in past two weeks. 1 = None, 2 = Once, 3 = Twice.

The rate of monthly substance use is shown in Table 2. The pattern of use mirrored that of annual use. On average, medical students reported an average consumption of 2.67 (*SD *= 3.25) alcoholic beverages per week. One third (33.8%, *n *= 290) of medical students reported consuming five or more drinks in one sitting in the past two weeks.

To examine potential differences in consumption based on gender, race, and sexual orientation, we ran a series of independent sample t-tests. Significant findings indicated that in comparison to female peers, male medical students drank more alcoholic beverages per week, *t*(434.00) = 5.93, *p *< .001, *d* = 0.57; consumed five or more alcoholic drinks more often, *t*(495.01) = 4.86, *p *< .001, *d *= 0.44; consumed alcohol more often in the past 30 days, *t*(596.84) = 4.60, *p* < .001, *d* = 0.38 and in the past year, *t*(832) = 3.91, *p *< .001, *d *= 0.27; smoked tobacco more often in the past 30 days, *t*(353.58) = 3.19, *p *= .002, *d *= 0.34 and past year, *t*(406.42) = 4.41, *p *< .001, *d *= 0.44; and smoked marijuana more often in the past 30 days, *t*(439.79) = 2.57, *p *= .010, *d *= .25. Non-Caucasian students drank significantly fewer alcoholic beverages per week than Caucasian students, *t*(405.64) = 3.02, *p *= .003, *d* = –.30; and consumed alcohol less frequently than Caucasian students in the past month, *t*(852) = 2.89, *p =* .004, *d *= –.20; and year *t*(299.21) = 3.76, *p *= .005, *d *= –.43. Effect sizes for these differences were small to moderate. There were no differences based on sexual orientation of students.

We then performed an analysis of variance to determine whether prevalence of substance use differed by geographic location of institutions. There were significant differences for reported consumption of five or more drinks in one sitting in the previous two weeks, *F*(3, 783) = 6.282, *p* < .001, η^2^ = .024. Students attending institutions in the West reported significantly lower consumption of five or more drinks in one sitting (*M *= 1.22, *SD* = 0.62) when compared to students in the Northeast (*M *= 1.66, *SD* = 0.98, *p* < .001, *d* = −0.54) and the Midwest (*M *= 1.57, *SD* = 0.91, *p *= .003, *d* = −0.45).

### Perceptions

Four questions on the Core Alcohol and Drug Survey [11] examined student perceptions about their respective medical school alcohol and drug policies. Approximately half of the students (52.7%, *n *= 451) reported that their medical college had an alcohol and drug policy; 46.0% (*n* = 393) did not know whether their institution had such a policy and 1.2% (*n *= 10) said that their college had no policy. Nearly three quarters of students (71.9%, *n* = 615) did not know whether their institution’s alcohol and drug policies were enforced; 24.3% (*n* = 208) of students said policies were enforced and 3.6% (*n* = 31) said they were not.

Regarding school-based programming, resources, and attitudes, 70.4% of students (*n* = 602) stated they did not know whether their medical college had an alcohol and drug prevention program; 23.9% (*n* = 204) of students said their college did have a program, and 5.4% (*n *= 46) said their college did not. Finally, over half of the students (55.8%, *n* = 476) believed their medical college was concerned about the prevention of alcohol and drug use. About one third of students (30.6%, *n* = 262) did not know whether their medical college was concerned, and 13.5% (*n* = 115) reported their college was not concerned about alcohol and drug use.

### Consequences

**Figure 2.**
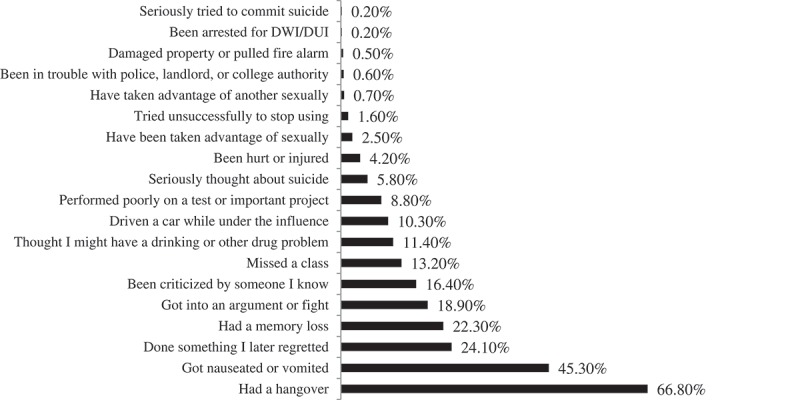
Consequences experienced by students in past year due to substance use.

Frequencies of health, mental health, cognitive, social, and academic consequences due to alcohol or drug use in the previous year are shown in Figure 2. The most common consequences were hangover (66.8%, *n* = 571), feeling nauseated or vomiting (45.3%, *n* = 387), and memory loss (22.3%, 191). Academic consequences included missing a class (13.2%, *n* = 113) and performing poorly on a test or important project (8.8%, *n* = 75). A considerable number of medical students reported driving while under the influence within the past year (10.3%, *n* = 88). A similar number of students thought they may have a drinking or drug use problem (11.4%, *n* = 97).

## Discussion

The purpose of this investigation was to assess the prevalence and consequences of substance use in US medical students and to examine medical students’ views of substance-use policies and programs provided at their institution. Our findings indicate that 91.3% and 26.2% of medical students consumed alcohol and used marijuana respectively in the past year. One third of medical students (33.8%) consumed five or more drinks in one sitting in the past two weeks. Consequences of student alcohol and drug use in this sample of medical students included but were not limited to interpersonal altercations, serious suicidal ideation, cognitive deficits, compromised academic performance, and driving under the influence of substances.

In the most recent comprehensive study conducted 25 years ago, students reported using the following substances within 30 days of data collection: alcohol (87.5%), marijuana (10%), and cigarettes (10%) among many others with lower prevalence [7]. Students in our study reported consuming alcohol (91.3%) and marijuana (11.7%) at slightly higher rates, although the use of tobacco (6.8%) slightly decreased. Comparatively, those in the general population with ages ranging from 18–25 and 26 and older reported rates of alcohol use within the past month at 59.6% and 56.5% respectively [1], which is notably lower than medical students of similar ages in this investigation. Consistent with other studies, we found that male medical students tend to consume significantly more alcohol than their female peers [4]. This pattern remained when comparing our sample to age matched peers in the general population; both male (87.8%) and female medical (83.9%) students in the current sample consumed alcohol in the past 30 days at higher rates than male (72.8% of 21–25 year olds; 69.3% of 26–34 year olds) and female (65.4% of 21–25 year olds, 59.7% of 26–34 year olds) respondents in the general population [18].

Our finding that one third of medical students had five or more drinks in a sitting within the past two weeks mirrors previous data on alcohol consumption of medical students [19], but revealed a much lower rate than that found in a recent single-institution study that included a lower threshold for at-risk drinking [5]. Note that this finding also serves as a conservative estimate, as our threshold for at-risk drinking was five drinks per sitting (rather than four) for female respondents. We used this threshold to maintain the psychometric support of the Core Alcohol and Drug Survey [11]. Thus, our study may have underestimated the rate of at-risk drinking due to our use of a conservative measure.

Similar rates of marijuana use have also recently been reported [5]. This finding is concerning because marijuana potency is stronger than it was 25 years ago [20]. Although our study suggests consumption has not drastically changed over the past 25 years, research suggests that heavy drinking and marijuana use patterns may be higher at individual medical schools than that found in national samples [5,7,21].

Our findings provide empirical evidence that medical students frequently experience personal and academic consequences of substance use during medical school. Serious consequences associated with alcohol and drug use were reported: 22.3% experienced memory loss, 5.8% had seriously considered suicide, 24.1% reported doing something they later regretted, 18.9% reported getting into an argument or fight, and more than 10% admitted that they had driven while under the influence of alcohol or drugs. Such findings are disconcerting, as this group of consequences suggests that substance use in medical students frequently precipitates severe lapses in professionalism [4,8,22,23], and too often endangers the lives of not only the student who is using but the lives of peers and others they may care for in the future.

Three quarters of students in our study did not know whether substance policies were enforced, and nearly half of the participants reported feeling that the school is either neutral or unconcerned about substance use and abuse at their medical school. Despite decades of alcohol and drug use awareness campaigns that often take place as early as middle school [24], these findings reflect a nearly two-fold increase over the past 25 years in the proportion of students who are unaware of whether or not their institution has an alcohol and drug policy [7]. In addition to re-examining, redesigning, and enforcing tighter boundaries pertaining to the use of alcohol and drugs among medical students, medical schools may want to prioritize students’ mental and physical health by creating a safe environment to talk about substance use and its consequences without the fear of backlash, and to both provide and promote services and resources for students in need of help.

Researchers have suggested that medical students who frequently use drugs or drink to excess during medical school, which includes after-exam partying, are at risk for developing a pattern of habitual use, and that this may foreshadow future consequences in residency and beyond [4,5,22,23]. The tendency to use substances to achieve mind altering effects or temporary relief from the academic demands of medical school makes these students susceptible to engaging in a pattern of risky social drinking in the future; such behavior has also been shown to affect professional decision making, such as providing alcohol counseling referrals to patients less often than those who do not drink [19].

Research indicates that substance-use behaviors predictive of substance-use disorders occur well before students enter medical school [6,8]. With early intervention and monitoring, treatment of substance use is often successful [25]. Thus, providing screening for students is a crucial area for medical education development, and one that many students are in fact already receiving during their education [26]. Curricula addressing the personal and professional consequences of substance use, as well as processes for screening, intervening, and treating substance-use disorders provide an avenue for disseminating institutional concern about the substances used by medical students, as well as alternatives to substance use. Staff and faculty may also benefit through education on the signs of medical student substance use, in addition to training on how to intervene when needed. Guided interpersonal interactions that include screening, brief intervention and referral to treatment [27], and digital resources have also been shown to be effective [28–30]. Our findings reinforce the need for all clinicians who treat medical students to obtain a complete substance-use history, including history of consequences and the possibility of co-occurring mood and anxiety disorders.

Although one would assume that medical schools have alcohol and drug use policies, many students shared that they were not aware of them. Only thirty percent of our participants were aware of whether or not substance-use prevention programs were available at their institution, even though they felt that their peers were involved in substance use at alarmingly high rates. Increasing both faculty and student awareness of guidelines, prevention, intervention, and treatment programs available to medical students is an important step for each institution to consider. Information on substance-use policies and risks can be conveyed in multiple ways to students at first-year orientation meetings, in ongoing wellness programs, in human behavior and neuroscience curricula, and in ongoing discussions between faculty and students on the risks of substance use during medical school. In addition to raising student awareness of the risks of substance use during medical education, educating faculty about medical students’ unique vulnerabilities to substance use during medical school is equally important. Ultimately, it is educational leadership that will direct meaningful solutions to the problem of medical student substance use.

### Limitations

A number of studies have supported the validity of anonymous self-report data [31–33]. Our survey relied on self-report responses from several pools of students, thus social desirability factors and recall bias may affect the generalizability of the findings. Although we used a respondent-driven sampling method that recruited students from 49 medical institutions throughout the US, our ability to capture a fully representative sample of the US medical student population remains limited. Our sample included a higher proportion of Caucasian and Native American students, and a lower proportion of Asian, African American, and Latino/a students. Female students were over represented in our sample, yet other recent surveys of substance use in US medical students have similarly reported a higher proportion of female respondents [5,34]. It is likely that the overall rates found in this study may underestimate the prevalence substance use among medical students due to this somewhat skewed sample.

Our results may also be affected by non-response bias, as students engaging in high-risk substance-use behaviors may have chosen not to participate in the study. Additionally, recall bias may have occurred due to the 12-month time span, of which students were asked to report. Although it is possible that students might have under-reported their substance use thereby introducing self-selection bias, the fact that our study was confidential and administered by an institution separate from most students’ home institution may have motivated students to answer more honestly than they would have if the research had been done within their institution. Despite these limitations, our results provide an updated look at the prevalence and consequences of medical student alcohol and drug use in the United States and provide medical educators a foundation on which to further assess the impact of substance-use intervention during medical education.

## Conclusion

Our findings suggest that nearly all medical student respondents had consumed alcohol in the past year, over a quarter of students used marijuana in the past year, and a third consumed five or more drinks in a sitting in the past two weeks. Our study is consistent with previous research suggesting that many medical students use illicit drugs [6,7] and drink heavily [4,5]. Our findings also indicate that substance use during medical school often impacts students’ personal and professional lives. Students routinely experience a number of consequences after using substances, including violence, suicidal ideation, driving under the influence, and cognitive impairment. Most medical students were not aware of their institution’s substance-use prevention programs, did not know if their institution’s substance-use policies were enforced, and felt that their medical school was either neutral or not concerned about medical student substance-use disorders. Although there has been some progress in characterizing medical student alcohol use, less is known about the factors surrounding medical students’ use of other substances. Updated, comprehensive studies on the patterns of medical student substance use are needed if we are to make the necessary changes needed to effectively prevent substance-use disorders among medical students and support those who are in need of help.
